# Safety and Efficacy of Dietary Epigallocatechin Gallate Supplementation in Attenuating Hypertension via Its Modulatory Activities on the Intrarenal Renin–Angiotensin System in Spontaneously Hypertensive Rats

**DOI:** 10.3390/nu14214605

**Published:** 2022-11-01

**Authors:** Kim Wai Parn, Wei Chih Ling, Jin Han Chin, Siew-Keah Lee

**Affiliations:** 1 M. Kandiah Faculty of Medicine and Health Sciences, Universiti Tunku Abdul Rahman, Kajang 43000, Malaysia; 2 Faculty of Medicine, Bioscience & Nursing, MAHSA University, Jenjarom 42610, Malaysia

**Keywords:** EGCG, hepatotoxicity, NOAEL, renin—angiotensin system, spontaneously hypertensive rats

## Abstract

This study aimed to identify the no-observed-adverse-effect level (NOAEL) of dietary epigallocatechin gallate (EGCG) supplementation and its possible antihypertensive and nutrigenomics effects in modulating intrarenal renin-angiotensin system (RAS) gene expression in spontaneously hypertensive rats (SHR). EGCG (50, 250, 500 or 1000 mg/kg b.w. i.g., once daily) was administered to SHR for 28 days. All the SHR survived with no signs of systemic toxicity. Increased alanine aminotransferase (ALT), aspartate aminotransferase (AST) and thiobarbituric acid reactive substances (TBARS) were evident in SHR supplemented with 500 and 1000 mg/kg b.w. but not in those supplemented with lower doses of EGCG. Subsequently, the NOAEL of EGCG was established at 250 mg/kg b.w., and the same protocol was replicated to assess its effects on blood pressure and renal RAS-related genes in SHR. The systolic blood pressure (SBP) of the EGCG group was consistently lower than the control group. The mRNA levels of cortical *Agtr2* and *Ace2* and medullary *Agtr2, Ace and Mas1* were upregulated while medullary *Ren* was downregulated in EGCG group. Statistical analysis showed that SBP reduction was associated with the changes in medullary *Agtr2*, *Ace*, and *Ren*. Dietary EGCG supplementation exhibits antihypertensive and nutrigenomics effects through activation of intrarenal *Ace* and *Agtr2* and suppression of *Ren* mediators, while a high dose of EGCG induced liver damage in SHR. In future clinical studies, liver damage biomarkers should be closely monitored to further establish the safety of the long-term use of EGCG.

## 1. Introduction

Dysregulation of the renin—angiotensin system (RAS), the main blood pressure regulator, is associated with several pathological conditions which include hypertension, atherosclerosis, heart and kidney diseases. In recent decades, research has been focusing on overactivity of the classical RAS in the development of hypertension; the roles of the counter-regulatory RAS in promoting cardiovascular health have gained popularity in recent decades [1].

The angiotensin-converting enzyme/angiotensin II/angiotensin II type 1 receptor [ACE/Ang II/AT1R] and prorenin/(pro)renin receptor are the two axes in classical RAS in which activation of these axes leads to vasoconstriction and subsequently elevated systemic blood pressure. Activation of the counter-regulatory arm via ACE/AngII/angiotensin II type 2 receptor (AT2R) or angiotensin-converting enzyme 2/angiotensin-(1–)/Mas receptor [ACE2/Ang-(1–7)/MasR] axes counteract the action of the classical arm, leading to vasodilation and reduction in systemic blood pressure [1].

The RAS can operate as an endocrine (commonly knowns as circulating or systemic RAS) and autocrine/paracrine (commonly known as local, or tissue-based RAS) in the vasculature, brain, heart, kidney, etc. The regulatory significance of local RAS components such as renin, angiotensinogen, ACE, Ang I and II is well recognized [2]. It has been demonstrated that systemic RAS involves short-term hemodynamic effects regulation while local RAS involves structural rebuilding of the vascular wall, brain, heart or kidney [3]. Studies on transgenic animal models have uncovered that intrarenal RAS is implicated in the pathogenesis of hypertension and renal diseases, and manipulation of intrarenal RAS mediators is associated with blood pressure reduction [4]. 

Overactivation of the classical RAS is frequently found in hypertensive individuals, usually evidenced by increased Ang II and AT1R levels [5]. Uses of RAS-blockade agents, i.e., ACE inhibitors and angiotensin receptor blockers which aim to suppress Ang II production are the therapeutic strategies in the management of hypertension. However, given limitations in using the current synthetic RAS inhibitors that cause various undesirable effects and intolerance in some patient populations [6,7], the need on exploration of novel RAS modulators is indeed in demand. Recent studies have showed that targeting counter-regulatory RAS mediators, e.g., AT2R and ACE2 could be a novel approach in managing hypertension [8,9].

Green tea (*Camellia sinensis*) is consumed worldwide and has been considered as a health-promoting beverage for centuries. In recent decades, green tea polyphenols have been reported scientifically to have therapeutic effects in cardiovascular diseases [10], neurodegenerative diseases [11], type 2 diabetes mellitus [12] and even in the prevention of cancers [13]. In addition to conventional tea infusion, concentrated green tea extract or pure epigallocatechin gallate (EGCG) prepared in oral capsule form are available widely as nutritional supplements and has become a popular choice for consumers [14].

EGCG, a polyphenol predominantly found in green tea catechin is a potent anti-oxidative and anti-inflammatory molecule and has been recommended as a health supplement for cardiac health. Uses of green tea or green tea extract in relation to its blood pressure-lowering effect have been demonstrated [14]. In in vivo studies, it has been demonstrated that the blood pressure-lowering effect of EGCG is in a time and dose-dependent manner [15,16]. Exploratory research has showed that EGCG is a potential blockage agent in inhibiting ACE and renin activities in in silico and in vitro studies [17,18]. Nevertheless, there is lack of scientific evidence in correlating the nutrigenomics effects of EGCG on RAS expression with its blood pressure-lowering effect. 

EGCG is in general considered to be safe and well-tolerated in humans and it is widely available in the market as a health supplement [19]. A recent comprehensive investigation by the European Food Safety Authority concluded that (i) catechins from green tea infusion prepared traditionally are in general considered to be safe (ii) rare cases of liver injury have been reported after consumption of green tea infusions/EGCG supplements, most probably due to an idiosyncratic reaction (iii) intake of doses equal or above 800 mg EGCG/day as a food supplement has been shown to induce a statistically significant increase of serum transaminases in treated subjects compared to control [19]. Nevertheless, these reports did not elucidate the mechanism underlying the hepatoxicity effect of EGCG, and there is still lacking scientific evidence in addressing the association of EGCG ingestion with organ damage.

This present study hypothesizes that higher doses of EGCG with longer supplementation period may be beneficial from greater blood pressure reduction in a genetically hypertensive rodent model, spontaneously hypertensive rats (SHR). With the consideration of potential toxic effect of EGCG, this study, therefore, aims to establish the no-observed-adverse-effect level (NOAEL) of EGCG before proceeding to examine the beneficial effects of EGCG. The second phase of the study aims to investigate the efficacy of EGCG in attenuating hypertension via its modulatory activities on the transcriptional levels of intrarenal RAS in SHR. SHR is selected as the study model as it has been established that the intrarenal RAS is inappropriately activated and attributed to the development of hypertension and renal damage [4].

## 2. Materials and Methods

### 2.1. Chemicals

EGCG (purity > 94%, Lot Number: M19023A01) was purchased from Taiyo GmbH (Gevelsberg, Germany). EGCG solution was prepared using phosphate-buffered saline as the vehicle solution. 2,7-dichlorofluorescein diacetate (DCFDA) was obtained from Canvax (Córdoba, Spain). Bradford reagent was purchased from HiMedia (Thane West, India). 

### 2.2. Experimental Animals

Male SHR aged 8–10 weeks old were procured from the University of Malaya, Malaysia and housed in the Animal Housing Facility, Universiti Tunku Abdul Rahman (UTAR), Malaysia. The animals were housed with alternate 12 h light–dark cycle at 22 ± 2 °C with ad libitum access to standard chow and tap water. The animals were acclimatized for both handlings and blood pressure measurement at least 2 weeks prior to the commencement of the actual experiment. Blood pressure of the animals were screened using the indirect tail-cuff plethysmography method (CODA^®^ Monitor, Kent Scientific Corporation, Torrington, CT, USA), and those with systolic blood pressure (SBP) lower than 160 mmHg were excluded from the study.

### 2.3. Phase I: Determination of NOAEL of EGCG

Repeated dose 28-day oral toxicity study of EGCG was conducted in accordance with the organisation for economic co-operation and development (OECD) Test Guideline 407. A total of 30 male SHR aged 12 weeks with SBP > 160 mmHg were randomly divided into 5 groups (*n* = 6 per group) and were supplemented with EGCG at 0 (placebo, was given the vehicle solution), 50, 250, 500 or 1000 mg/kg body weight (once a day, via oral gavage) for a period of consecutive 28 days (day 1 to day 28). The human equivalency dose (HED) for 50, 250, 500, and 1000 mg/kg EGCG in rats are 486, 2430, 4860, and 9720 mg EGCG, respectively through conversion based on the body surface area [20] which corresponds to approximately 3–57 cups of green tea by assuming each cups contained 2.5 g green tea leaves in 250 mL boiling water [21].

The body weight, food, and water intakes were measured daily. Cage-side observation for behaviour changes and possible toxicity events (inactivity, aggressive, porphyrin staining, eye or skin inflammation, piloerection, reduced appetite, diarrhoea, breathing difficulties, and mortality) was performed at the 30 min and 4 h post-administration of EGCG throughout the experimental period (Table 1). 

On day 29, the rats were euthanized via inhalation of excess carbon dioxide followed by cervical dislocation to confirm terminal anaesthesia. Blood was drawn from the inferior vena cava and stored in heparinized-tube, centrifuged at 1000× *g* for 15 min at 4 °C to collect plasma for further biochemical analysis [alanine aminotransferase (ALT); aspartate aminotransferase (AST), urea, creatinine].

The rats were then dissected and the thoracic (heart and lungs) and abdominal organs (liver, stomach, spleen, small intestine, large intestine, kidneys) were observed macroscopically for the presence of gross lesions, changes in size and external appearance. The organs were then removed, blot dried and weighted with a digital weighing scale. Relative organ weight was calculated using the following formula: relative organ weight (g/100 g b.w.) = organ weight (g)/100 g of body weight. Liver tissues were cryopreserved using liquid nitrogen and stored at −80 °C for further biochemical analysis [thiobarbituric acid reactive substances (TBARS), reactive oxygen species (ROS), caspase-3 levels]. 

#### 2.3.1. Determination of Biomarkers of Liver and Kidney Injuries

Plasma ALT and AST levels were determined by using commercially available assay kits (Cayman Chemical, Ann Arbor, MI, USA). Plasma creatinine and urea levels were determined by using Biolis 24i Premium automated biochemical analyzer (Tokyo, Japan).

#### 2.3.2. Determination of the Hepatic Oxidative and Apoptosis Status

The 10% (*w*/*v*) liver tissue homogenate was prepared by adding 9 volumes of ice-cold 40 mM Tris-HCl buffer, pH 7.4 and homogenate on ice using the Omni tissue homogenizer (Omni International, Kennesaw, GA, USA). The 10% tissue homogenate was centrifuged at 10,000× *g*, 15 min, at 4 °C and the supernatant was collected and stored at −80 °C for further determination of levels of TBARS, ROS, and caspase-3.

Liver TBARS and caspase-3 levels were measured using commercially available assay kits (Elabscience, Wuhan, China). Liver ROS level was measured using commercially available assay kit (Canvax, Córdoba, Spain) with modification as follows: 100 μL of 10% liver tissue homogenate was added with 100 μL 10 μM DCFDA followed by 30 min incubation at 37 °C. The intensity of fluorescence signal was measured at Ex/Em = 485/530 nm using the Spark^®^ multimode microplate reader (Tecan, Zürich, Switzerland). Total protein was quantified using the Bradford method for the normalization of ROS and caspase-3 levels.

### 2.4. Phase II: Determination of Antihypertensive Efficacy of Oral EGCG and Its Nutrigenomics Effects on Expression of RAS-Related Genes in SHR

The NOAEL of EGCG (250 mg/kg b.w.), or vehicle were given via oral gavage to the male SHR aged 12 weeks with SBP > 160 mmHg (*n* = 6 per group) once daily for 28 consecutive days. The cage-side observation was performed throughout the supplementation period as per the protocol stated in the Phase I study. On day 29, the SHR were euthanized and dissected, observed for the presence of gross lesions, changes in size and external appearance of the thoracic and abdominal organs as stated in the phase I study. The kidneys were harvested, washed, divided into renal cortex and medulla specimens and stored in RNAlater^TM^ solution for gene expression study. 

#### 2.4.1. Blood Pressure Measurement

Blood pressure of the SHR were measured on day 0 (one day prior to commencement of EGCG administration), 7, 14, 21, and 28 of the supplementation period using the indirect tail-cuff volume pressure recording method (CODA, Kent Scientific, Torrington, CT, USA) as stated in our previous study [16].

#### 2.4.2. Measurement of mRNA Expression of RAS-Related Genes

The total RNA of the renal cortex and medulla were isolated using Monarch^®^ Total RNA Miniprep Kit (New England Biolabs, Ipswich, MA, USA) according to the manufacturer’s protocol. The purity and concentration of extracted RNA were assessed through Nanodrop^TM^ 2000 spectrophotometer (Thermo Fisher Scientific, Wilmington, NC, USA). The RNA integrity was examined using the bleach gel method [22]. cDNA was synthesized using High-Capacity cDNA Reverse Transcription Kit (Applied Biosystems, Waltham, MA, USA). Primer sequences were obtained from previous studies and verified via Primer-BLAST software (Table 2). The real-time PCR was performed using Luna^®^ Universal qPCR Master Mix (New England Biolabs, Ipswich, MA, USA) on the StepOnePlus^TM^ Real-Time PCR System (Applied Biosystems, Waltham, MA, USA). The thermocycler condition for all PCR reactions was as follows: 95 °C for 1 min, 40 cycles at 95 °C for 15 s and 60 °C for 30 s, followed by the melt curve analysis. The mRNA expression level was normalized to *Gapdh* expression. The relative mRNA expression was calculated using the 2^−ΔΔCt^ method.

### 2.5. Statistical Analysis

The data were presented as mean ± standard deviation (SD). Statistical analyses were conducted using IBM SPSS Statistics for Windows, version 26 (IBM Corp., Armonk, NY, USA). Continuous data were checked with the normality test and Levene’s test, and then continuous data with confirmed normal distribution were analyzed using one-way or two-way ANOVA followed by Dunnett’s post hoc test for multiple comparisons, or independent *t*-test for 2-group comparisons. Pearson correlation test was conducted to examine the relationship between blood pressure parameters and RAS-related genes that were significantly affected by EGCG supplementation. A *p*-value < 0.05 was considered statistically significant.

## 3. Results

### 3.1. Cage-Side Observation and Gross Anatomy of the Thoracic and Abdominal Organs and Their Relative Organ Weights

All the rats survived until the end of the supplementation period. There are no changes in the body weight, food, and water intake in EGCG groups compared with the placebo group (Table 3). Cage-side observation showed the absence of toxic signs in all the groups (Table 4). There were neither changes in the gross anatomy of the thoracic and abdominal organs nor the relative organ weights between EGCG and placebo groups (Table 5).

### 3.2. Effect of EGCG on Plasma and Hepatic Biochemical Parameters

**Figure 1:**
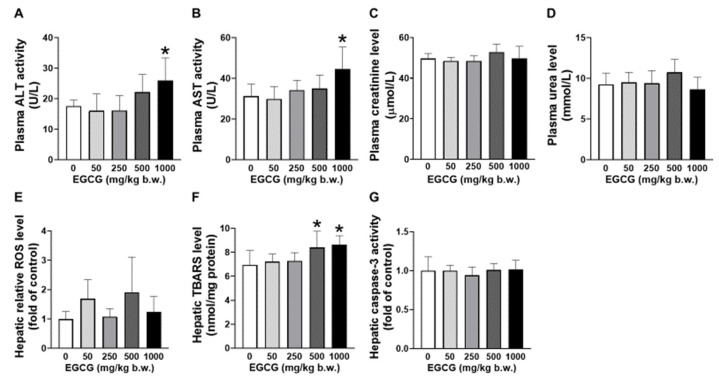
Effect of EGCG at different concentrations on the plasma (**A**) AST, (**B**) ALT, (**C**) creatinine, (**D**) urea and hepatic (**E**) ROS, (**F**) TBARS, and (**G**) caspase-3 of SHR. Data were expressed as mean ± SD, *n* = 6. * *p* < 0.05 vs. vehicle, one-way ANOVA followed by Dunnett’s test. ALT: alanine aminotransferase, AST: aspartate aminotransferase, ROS: reactive oxygen species, TBARS: thiobarbituric acid reactive substances.

Elevated plasma ALT and AST levels were found in SHR supplemented with 1000 mg/kg EGCG compared to the placebo group (Figure 1A,B). EGCG supplementation did not cause significant changes in the levels of plasma creatinine and urea (Figure 1C,D). Increased hepatic TBARS was noticed in SHR supplemented with 500 and 1000 mg/kg of EGCG (Figure 1E). No changes in hepatic ROS and caspase-3 levels among the supplemented and placebo groups (Figure 1F,G).

### 3.3. Effect of EGCG on Blood Pressure Parameters

**Figure 2:**
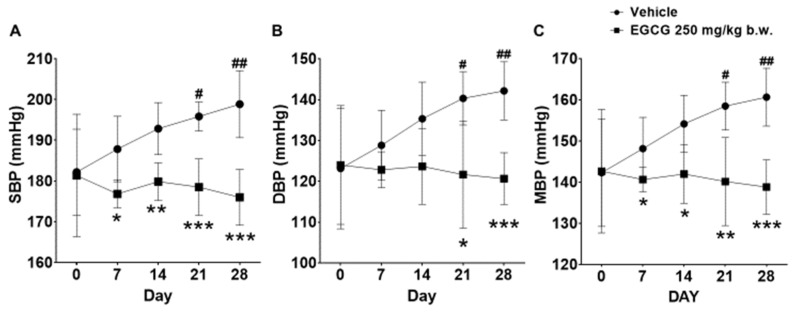
Effect of 28 days EGCG treatment on the (**A**) SBP, (**B**) DBP, and (**C**) MBP of the SHR. Data were expressed as mean ± SD, *n* = 6. # *p* < 0.05, ## *p* < 0.01 vs. day 0; * *p* < 0.05, ** *p* < 0.01, *** *p* < 0.001 vs. vehicle, two-way ANOVA followed by Dunnett’s test. SBP: systolic blood pressure, DBP: diastolic blood pressure, MBP: mean blood pressure.

SHR supplemented with 250 mg/kg EGCG showed significant reductions in SBP, diastolic blood pressure (DBP), and mean blood pressure (MBP) by 23, 21, and 22 mmHg, respectively at day 28 compared with the placebo group. The SBP and MBP reduction was evident from day 7 onwards, while DBP reduction was apparent on day 21 and day 28 (Figure 2).

### 3.4. Effect of EGCG on the mRNA Expression of Cortical and Medullary RAS-Related Genes

**Figure 3:**
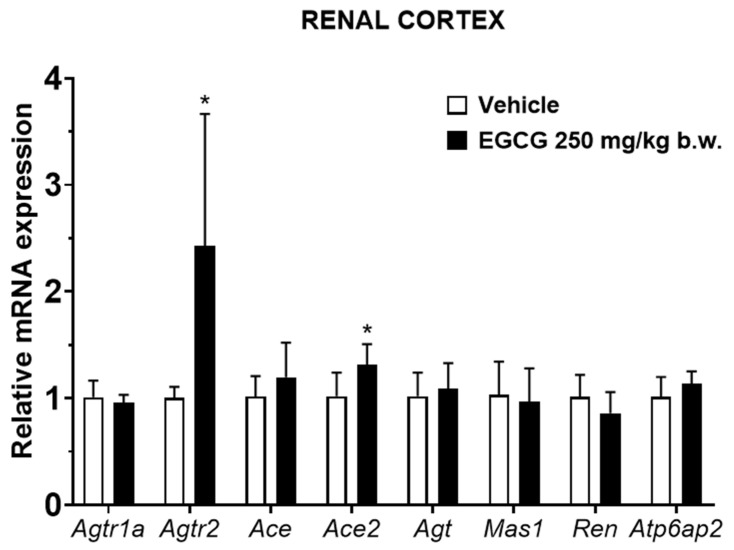
Relative mRNA expression of RAS related genes in renal cortex. Data were normalized to the *Gadph* gene expression and expressed as mean ± SD, *n* = 6. * *p* < 0.05 vs. vehicle, Student’s *t*-test.

**Figure 4:**
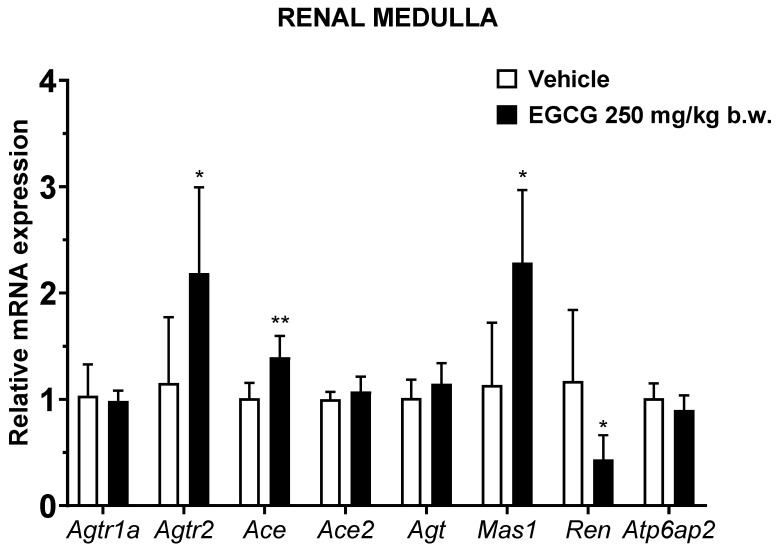
Relative mRNA expression of RAS related genes in renal medulla. Data were normalized to the *Gadph* gene expression and expressed as mean ± SD, *n* = 6. * *p* < 0.05, ** *p* < 0.01 vs. vehicle, Student’s *t*-test.

EGCG significantly upregulated renal cortical *Atgr2* and *Ace2* and medullary *Agtr2*, *Ace*, and *Mas1* levels, while it significantly downregulated medullary *Ren* mRNA level in EGCG group (Figure 3 and Figure 4).

### 3.5. Statistical Correlation between Blood Pressure Parameters and RAS-Related Genes

SBP reduction in EGCG group was significantly correlated with the upregulated medullary *Agtr2* and *Ace* and downregulated *Ren* levels. Similar non-significant trending of associations was observed in DBP and MBP and the related genes (Table 6).

## 4. Discussion

In general, 4 weeks of subacute oral administration of EGCG at 500 mg/kg b.w. increases the hepatocellular lipid peroxidation which reflected by the elevated TBARS levels. When EGCG is given at a higher dose (1000 mg/kg b.w.), it causes further hepatocellular injury indicated by the increased level of plasma transaminases (ALT and AST). No evidence of hepatocyte apoptosis via observation of the unchanged level of the apoptosis biomarker (caspase-3) suggests that the liver damage could be at a mild stage. Therefore, this study concludes that 4 weeks of EGCG supplementation at a dose of 250 mg/kg b.w. (HED = 2430 mg) does not cause statistically significant increase in the frequency of adverse effects in SHR. Further molecular study shows that the blood pressure-lowering effect of EGCG is associated with the upregulated renal ACE and AT2R expressions. Increased activation of AT2R further suppresses renin expression via the negative feedback loop, inhibits the entire systemic and local classical arm of RAS, and results in blood pressure reduction. 

Inadequate evidence on EGCG hepatotoxicity in the previous literature could be due to (i) EGCG leads to mild liver injury that could not be identified via cage-side observation or necropsy (ii) lack of awareness among the researchers on the potential toxic effect of EGCG. The present study shows that the 500–1000 mg/kg b.w. i.g. EGCG-induced hepatotoxicity is at a mild stage and not detectable through cage-side observation and necropsy findings. Previous toxicology studies revealed that a single dose of oral 2000 mg/kg b.w. EGCG (93% purity) was lethal to Wistar rats, while supplementation of 13 weeks of repeated dose of 500 mg/kg b.w. EGCG-containing diet (77% purity) did not cause deaths or observable clinical signs, as well as changes in hematology profile, liver (ALT, AST) and kidney (blood urea nitrogen, creatinine injury biomarkers at the end of the supplementation period and 4-week of recovery period in Sprague Dawley rats [23]. Our study reveals that supplementation of 500 mg/kg b.w. EGCG with purity > 94% for 4 weeks leads to augmented ALT and AST levels in SHR. Non-dosing recovery phase was not included in this present study mainly to increase the sensitivity in detecting supplementation-related injury as well as to avoid the “masking effect” of cellular self-recovery as noticed in a previous study [24]. A lower dose of EGCG (150 mg/kg b.w.) administered via intraperitoneal route showed significant nephrotoxicity in male Swiss albino mice [25] while this present study showed that orally administered EGCG up to 1000 mg/kg b.w. did not cause changes in kidney injury biomarkers in genetically hypertensive SHR. Inconsistent findings on the safety profile of EGCG among studies could be due to variation in (i) purity, duration and route of administration of the administered EGCG (ii) degree of susceptibility to EGCG due to different genetic make-up, pathological conditions or feeding status [23,24,25,26]. 

The underlying mechanisms on how EGCG causes organ damage particularly hepatoxicity are not well studied. Lambert et al. [26] have demonstrated that EGCG (500–1500 mg/kg b.w., i.g.)-induced hepatoxicity was associated with induction of hepatic oxidative stress (with elevated oxidative stress markers, TBARS, ALT, AST), and these effects are in a time- and dose-dependent manner in mice. Wang et al. [27] reported that EGCG triggered hepatoxicity through transcriptional inhibition of the major intracellular antioxidant enzymes, i.e., superoxide dismutase, catalase and glutathione peroxidative and subsequently led to excessive build-up of ROS and cellular damage, in addition to suppression of nuclear factor erythroid 2-related factor 2 pathway via downregulation of DJ-1 in mice.

This present study showed that antihypertensive effects of EGCG is in a cumulative time-dependent manner. EGCG suppressed the elevation of systolic blood pressure progressively at −11, 13, 17 and 23 mmHg (5.9%, 6.7%, 8.7%, and 11.6%) following 1,2,3, and 4 weeks of supplementation. These blood pressure lowering effects are significantly associated with the changes in the transcriptional levels of intrarenal *Ren*, *Ace* and *Agtr2*. Nevertheless, the causal relationship between changes in blood pressure and gene expression cannot be established in this present study. This study further postulates that activation of local ACE/Ang II/AT2R results in systemic blood pressure reduction, and the Ang II production is independent from the actions of the decreased renin shown in this study. 

In the classical RAS, renin cleaves angiotensinogen to Ang I, and is subsequently catalyzed by ACE to produce Ang II. On the other hand, (pro)renin receptors, tonin, cathepsin D and chymase are also involved in Ang II production [1]. The importance of these alternative pathways in Ang II production remains unelucidated, however, it has been associated with activation of the local AT1R and AT2R [1]. Activation of intrarenal Ang II/AT1R leads to vasoconstriction, sodium and water retention, tissue inflammation and fibrosis and oxidative stress [28]. While activation of Ang II/AT2R counteracts Ang II/AT1R biological effects by promoting vasodilation and natriuresis, reducing tissue inflammation, and inhibiting cell proliferation and differentiation [29]. Moreover, intrarenal Ang II/AT2R binding triggers the production of nitric oxide, bradykinin and prostaglandins and subsequently leads to vasodilatation and blood pressure reduction [29]. With the evidence of upregulated AT2R but insignificant changes of the AT1R abundance found in this present study, this study proposes that binding effects of Ang II/AT2R overtaking those triggered by Ang II/AT1R complex and ultimately results in blood pressure reduction in EGCG supplemented SHR. In addition, upregulated AT2R might form heterodimerization with AT1R and attenuates functionality of AngII/AT1R binding [30]. 

Reduced renal renin synthesis in EGCG supplemented SHR could be due to (i) the direct effect of EGCG on renin transcriptional levels or (ii) the negative feedback loop of AT2R activation. It has been demonstrated that green tea extract supplementation reduced renin expression in chronic cyclosporin-treated rats [31] while in vitro study has shown that EGCG is an effective agent in inhibiting renin activities [17]. Another group of research team has demonstrated that activation of AT2R suppressed renin synthesis via negative feedback mechanism [32]. In addition to its action in generation of Ang II, studies have demonstrated that renin itself may directly trigger cellular effects via activation of p38 mitogen-activated protein kinase that subsequently may lead to vascular damage and raised blood pressure [33]. Reduced renin undeniably can diminish RAS overactivation and inhibit further blood pressure elevation [3]. It is worth mentioning that *Ace2* and *Mas1* which are the mediators of the protective arm of the RAS are significantly upregulated in the EGCG-supplemented group, albeit statistical correlation test did not show its association with blood pressure reduction. ACE2 cleaves Ang II to Ang-(1–7), which is a ligand of MasR in promoting natriuresis and vasodilation [3]. A limitation of the study is that only mRNA levels of the RAS-related genes were assessed but not their protein expressions. 

## 5. Conclusions

The NOAEL of 28-day oral EGCG is 250 mg/kg b.w. in SHR, with HED of 2430 mg. Administration of EGCG beyond this NOAEL causes elevated hepatic lipid peroxidation and liver damage indicated by the elevated ALT and AST levels. EGCG at its NOAEL dose exhibits blood pressure lowering effects and it is associated with the activation of the intrarenal counter-regulatory axis (ACE/AngII/AT2R) and suppression of the classical axis (REN) gene expressions. In future clinical studies, liver damage biomarkers should be closely monitored to further establish the safety of the long-term use of EGCG.

## Figures and Tables

**Table 1:** Cage-side observation criteria.

Observations	Category	Explanation
General condition	Normal	Awake, active, reacts to stimulation
	Mild	Burrows in litter, hides, lies still but is startled when handled
	Severe	Immobile, little or no voluntary movement. Burrows/hides. Presses head against cage bottom. Vocalizes. Extremely afraid and/or aggressive when handled
Porphyrin staining and/or eye inflammation	Absent	No discoloration, clean and clear eyes
	Mild	Some porphyrin and/or discharge around eyes and nose
	Severe	Obvious porphyrin on ‘face’ and/or on legs and paws. Eye(s) closed, squints and/or discharge around eye(s)
Movements and posture	Normal	Normal coordination without any difficulty in movements
	Mild	Moderate in-coordination when animal is stimulated; hunched posture
	Severe	Marked in-coordination, head held at angle, hunched posture and/or back, does not support itself on all four limbs and/or paralysis
Piloerection	Absent	Fur smooth and well-groomed
	Mild	Moderate piloerection
	Severe	Severe piloerection, sticky and poorly groomed fur
Skin	Normal	Skin covered entirely with fur. No sores or other signs of injury
	Mild	Small sores or scabs, no infection; scratching (signs of itching)
	Severe	Bites or scratches itself or trauma from others. Signs of infection such as redness and/or pus or serious discharge; sticky and poorly groomed fur. Non-healing operation wounds or broken sutures
Appetite/Food and water intake	Normal	Normal appetite, eating and drinking regularly
	Mild	Reduced appetite, consume less food and water
	Severe	No interest in food and appears dehydrated
Defecation	Normal	Firm faecal boli with brown colour
	Mild	Faeces looser or harder than normal and/or abnormal colour
	Severe	Diarrhoea (excessive watery stool)/Constipation (no stool or very hard stool) and/or abnormal colour
Urination	Normal	Normal urine colour (pale yellow to yellowish) without any odour
	Abnormal	Abnormal urine colour and/or has strong odour
Breathing Difficulties	Absent	Normal respiration, not strained or wheezy
	Present	Breathes with open mouth, abdominal breathing or panting, crackle and/or gasping noises

**Table 2:** Primer sequences and amplicon sizes.

Gene	Accession Number	Primers		Amplicon Size (bp)	Encoded Protein
*Gapdh*	NM_017008.4	F:	ATGGGAAGCTGGTCATCAAC	221	Glyceraldehyde 3-phosphate dehydrogenase
		R:	GTGGTTCACACCCATCACAA		
*Agtr1a*	NM_030985.4	F:	CTGCCACATTCCCTGAGTTAAC	302	Angiotensin II receptor, type 1a
		R:	ATCACCACCAAGCTGTTTCC		
*Agtr2*	NM_012494.3	F:	TAATCTCAACGCAACTGGCACC	222	Angiotensin II receptor, type 2
		R:	GCCAAAAGGAGTAAGTCAGCCA		
*Ace*	NM_012544.1	F:	GCCCCCTGTACAAGTGTGAT	347	Angiotensin-converting enzyme
		R:	TAGGAAGAGCAGCACCCACT		
*Ace2*	NM_001012006.1	F:	CAGGAAGCTGAAGACCTGTCT	251	Angiotensin-converting enzyme 2
		R:	TTCAACTGTTTGTTCTTGTCTG		
*Agt*	NM_134432.2	F:	TTCAGGCCAAGACCTCCC	309	Angiotensinogen
		R:	CCAGCCGGGAGGTGCAGT		
*Mas1*	NM_012757.2	F:	GGCGGTCATCATCTTCATAGC	313	Mas receptor
		R:	CTTCTTCTTACTGCTGCCCAC		
*Ren*	NM_012642.4	F:	CACTCTTGTTGCTCTGGACCT	250	Renin
		R:	GGGGTACCAATGCCGATCTC		
*Atp6ap2*	NM_001007091.1	F:	CCGTGGCACCATGGCTGTGCT	204	(Pro)renin receptor
		R:	GCAAGCCCTGGCCAAGACAG		

**Table 3:** Body weight, food and water intakes.

Parameters	EGCG (mg/kg b.w.)				
	0	50	250	500	1000
Body weight (g)					
D0	262.9 ± 18.7	269.3 ± 17.2	273.8 ± 21.1	267.2 ± 18.7	266.2 ± 18.1
D7	275.4 ± 14.0	280.2 ± 19.1	283.9 ± 16.8	272.5 ± 16.6	275.1 ± 15.0
D14	285.2 ± 11.7	290.6 ± 18.7	291.8 ± 20.2	280.8 ± 18.4	282.5 ± 19.1
D21	293.6 ± 14.9	301.6 ± 21.4	302.6 ± 22.4	286.3 ± 13.9	295.4 ± 19.8
D28	305.8 ± 15.4	306.7 ± 23.7	309.6 ± 22.7	289.0 ± 13.3	296.4 ± 23.7
Food intake (g/100 g b.w.)					
D0	7.0 ± 1.6	7.8 ± 1.3	6.1 ± 1.2	6.2 ± 1.4	6.2 ± 0.8
D7	6.8 ± 1.3	7.7 ± 0.6	7.4 ± 1.2	6.8 ± 0.6	6.6 ± 0.3
D14	6.6 ± 1.2	7.0 ± 1.1	7.1 ± 0.8	6.9 ± 1.2	6.5 ± 0.7
D21	6.3 ± 0.6	6.5 ± 0.4	6.1 ± 0.5	6.4 ± 0.8	6.7 ± 0.2
D28	6.3 ± 1.3	6.4 ± 1.3	6.4 ± 1.1	6.2 ± 1.1	5.3 ± 0.6
Water intake (g/100 g b.w.)					
D0	8.6 ± 0.5	7.9 ± 1.9	8.8 ± 0.3	7.0 ± 0.1	9.8 ± 0.8
D7	8.5 ± 0.5	9.6 ± 0.2	9.4 ± 0.4	8.7 ± 0.2	8.5 ± 0.7
D14	8.6 ± 0.1	9.3 ± 0.9	9.5 ± 0.6	8.8 ± 0.1	9.2 ± 0.4
D21	8.3 ± 0.1	8.8 ± 0.4	9.1 ± 1.1	8.8 ± 0.7	9.5 ± 0.8
D28	8.0 ± 0.4	9.2 ± 1.3	9.6 ± 1.5	8.4 ± 1.1	7.8 ± 0.6

**Table 4:** Cage-side observation for behaviour changes and clinical signs.

Observations	EGCG (mg/kg b.w.)				
	0	50	250	500	1000
General condition	Normal	Normal	Normal	Normal	Normal
Porphyrin staining and/ or eye inflammation	Absent	Absent	Absent	Absent	Absent
Movements and posture	Normal	Normal	Normal	Normal	Normal
Piloerection	Absent	Absent	Absent	Absent	Absent
Skin	Normal	Normal	Normal	Normal	Normal
Appetite/Food and water intake	Normal	Normal	Normal	Normal	Normal
Defecation	Normal	Normal	Normal	Normal	Normal
Urination	Normal	Normal	Normal	Normal	Normal
Breathing Difficulties	Absent	Absent	Absent	Absent	Absent

**Table 5:** Relative organ weights.

Organ Index (g/100 g b.w.)	EGCG (mg/kg b.w.)				
	0	50	250	500	1000
Heart	0.4131 ± 0.0182	0.4147 ± 0.0260	0.4491 ± 0.0652	0.4429 ± 0.0267	0.4308 ± 0.0146
Lungs	0.4571 ± 0.0415	0.4856 ± 0.0371	0.4597 ± 0.0416	0.4969 ± 0.0441	0.5200 ± 0.0507
Liver	3.3948 ± 0.3451	3.5631 ± 0.1813	3.5441 ± 0.1658	3.5057 ± 0.2557	3.6100 ± 0.0819
Stomach	0.1891 ± 0.0096	0.1786 ± 0.0072	0.1958 ± 0.0116	0.2047 ± 0.0174	0.2024 ± 0.0141
Spleen	0.5151 ± 0.0964	0.4939 ± 0.0620	0.4415 ± 0.0316	0.4851 ± 0.0691	0.5047 ± 0.0790
Small intestine	1.7391 ± 0.3923	1.6474 ± 0.2582	1.3964 ± 0.2225	1.8305 ± 0.1406	1.6194 ± 0.1526
Large intestine	0.9406 ± 0.1173	0.8760 ± 0.0602	0.9205 ± 0.1570	0.9941 ± 0.1346	0.9963 ± 0.1378
Kidneys	0.7764 ± 0.0156	0.8076 ± 0.0416	0.7827 ± 0.0368	0.7902 ± 0.0319	0.8149 ± 0.0187

Relative organ weights were calculated through normalization of organ weight to body weight. Data were expressed as mean ± SD, *n* = 6.

**Table 6:** Pearson correlation analysis between RAS-related genes modulated by EGCG supplementation and blood pressure parameters.

RAS-Related Genes	SBP		DBP		MBP	
	*r*	*p*	*r*	*p*	*r*	*p*
Renal Cortex						
*Agtr2*	−0.497	0.100	−0.323	0.297	−0.400	0.198
*Ace2*	−0.283	0.373	0.084	0.794	−0.024	0.942
Renal Medulla						
*Agtr2*	−0.586 *	0.045	−0.545	0.067	−0.563	0.057
*Ace*	−0.585 *	0.046	−0.429	0.164	−0.494	0.102
*Mas1*	−0.506	0.093	−0.536	0.072	−0.521	0.082
*Ren*	0.584 *	0.046	0.349	0.267	0.441	0.151

SBP: systolic blood pressure, DBP: diastolic blood pressure, MBP: mean blood pressure. * *p* < 0.05.

## Data Availability

Data generated from this study can be obtained by emailing Siew-Keah Lee at leesiewkeah@utar.edu.my.

## References

[1] Nehme A., Zouein F.A., Zayeri Z.D., Zibara K. (2019). An Update on the Tissue Renin Angiotensin System and Its Role in Physiology and Pathology. J. Cardiovasc. Dev. Dis..

[2] Nehme A., Cerutti C., Dhaouadi N., Gustin M.P., Courand P.Y., Zibara K., Bricca G. (2015). Atlas of tissue renin-angiotensin-aldosterone system in human: A transcriptomic meta-analysis. Sci. Rep..

[3] Simko F., Hrenak J., Adamcova M., Paulis L. (2021). Renin-Angiotensin-Aldosterone System: Friend or Foe-The Matter of Balance. Insight on History, Therapeutic Implications and COVID-19 Interactions. Int. J. Mol. Sci..

[4] Kobori H., Nangaku M., Navar L.G., Nishiyama A. (2007). The intrarenal renin-angiotensin system: From physiology to the pathobiology of hypertension and kidney disease. Pharmacol. Rev..

[5] Szczepanska-Sadowska E., Czarzasta K., Cudnoch-Jedrzejewska A. (2018). Dysregulation of the Renin-Angiotensin System and the Vasopressinergic System Interactions in Cardiovascular Disorders. Curr. Hypertens. Rep..

[6] Marcum Z.A., Fried L.F. (2011). Aging and antihypertensive medication-related complications in the chronic kidney disease patient. Curr. Opin. Nephrol. Hypertens..

[7] Mishima E., Haruna Y., Arima H. (2019). Renin-angiotensin system inhibitors in hypertensive adults with non-diabetic CKD with or without proteinuria: A systematic review and meta-analysis of randomized trials. Hypertens. Res..

[8] Dai S.Y., Zhang Y.P., Peng W., Shen Y., He J.J. (2016). Central Infusion of Angiotensin II Type 2 Receptor Agonist Compound 21 Attenuates DOCA/NaCl-Induced Hypertension in Female Rats. Oxid. Med. Cell. Longev..

[9] Sartorio C.L., Pimentel E.B., Dos Santos R.L., Rouver W.N., Mill J.G. (2020). Acute hypotensive effect of diminazene aceturate in spontaneously hypertensive rats: Role of NO and Mas receptor. Clin. Exp. Pharmacol. Physiol..

[10] Liu G., Mi X.N., Zheng X.X., Xu Y.L., Lu J., Huang X.H. (2014). Effects of tea intake on blood pressure: A meta-analysis of randomised controlled trials. Br. J. Nutr..

[11] Lardner A.L. (2014). Neurobiological effects of the green tea constituent theanine and its potential role in the treatment of psychiatric and neurodegenerative disorders. Nutr. Neurosci..

[12] Wang X., Tian J., Jiang J., Li L., Ying X., Tian H., Nie M. (2014). Effects of green tea or green tea extract on insulin sensitivity and glycaemic control in populations at risk of type 2 diabetes mellitus: A systematic review and meta-analysis of randomised controlled trials. J. Hum. Nutr. Diet..

[13] Dou Q.P., Landis-Piwowar K.R., Chen D., Huo C., Wan S.B., Chan T.H. (2008). Green tea polyphenols as a natural tumour cell proteasome inhibitor. Inflammopharmacology.

[14] Xu R., Yang K., Ding J., Chen G. (2020). Effect of green tea supplementation on blood pressure: A systematic review and meta-analysis of randomized controlled trials. Medicine.

[15] Zhang Q., Hu L., Chen L., Li H., Wu J., Liu W., Zhang M., Yan G. (2018). (-)-Epigallocatechin-3-gallate, the major green tea catechin, regulates the desensitization of beta1 adrenoceptor via GRK2 in experimental heart failure. Inflammopharmacology.

[16] Tan H.J., Ling W.C., Chua A.L., Lee S.K. (2021). Oral epigallocatechin gallate reduces intestinal nadolol absorption via modulation of Oatp1a5 and Oct1 transcriptional levels in spontaneously hypertensive rats. Phytomedicine.

[17] Li F., Takahashi Y., Yamaki K. (2013). Inhibitory effect of catechin-related compounds on renin activity. Biomed. Res..

[18] Ke Z., Su Z., Zhang X., Cao Z., Ding Y., Cao L., Ding G., Wang Z., Liu H., Xiao W. (2017). Discovery of a potent angiotensin converting enzyme inhibitor via virtual screening. Bioorg. Med. Chem. Lett..

[19] Younes M., Aggett P., Aguilar F., Crebelli R., Dusemund B., Filipič M., Frutos M.J., Galtier P., Gott D., EFSA Panel on Food Additives and Nutrient Sources added to Food (ANS) (2018). Scientific opinion on the safety of green tea catechins. EFSA J..

[20] Nair A.B., Jacob S. (2016). A simple practice guide for dose conversion between animals and human. J. Basic Clin. Pharm..

[21] Haytowitz D.B., Wu X., Bhagwat S. (2018). USDA Database for the Flavonoid Content of Selected Foods, Release 3.3.

[22] Aranda P.S., LaJoie D.M., Jorcyk C.L. (2012). Bleach gel: A simple agarose gel for analyzing RNA quality. Electrophoresis.

[23] Isbrucker R.A., Edwards J.A., Wolz E., Davidovich A., Bausch J. (2006). Safety studies on epigallocatechin gallate (EGCG) preparations. Part 2: Dermal, acute and short-term toxicity studies. Food Chem. Toxicol..

[24] Ramachandran B., Jayavelu S., Murhekar K., Rajkumar T. (2016). Repeated dose studies with pure Epigallocatechin-3-gallate demonstrated dose and route dependant hepatotoxicity with associated dyslipidemia. Toxicol. Rep..

[25] Rasheed N.O., Ahmed L.A., Abdallah D.M., El-Sayeh B.M. (2017). Nephro-toxic effects of intraperitoneally injected EGCG in diabetic mice: Involvement of oxidative stress, inflammation and apoptosis. Sci. Rep..

[26] Lambert J.D., Kennett M.J., Sang S., Reuhl K.R., Ju J., Yang C.S. (2010). Hepatotoxicity of high oral dose (-)-epigallocatechin-3-gallate in mice. Food Chem. Toxicol..

[27] Wang D., Wang Y., Wan X., Yang C.S., Zhang J. (2015). Green tea polyphenol (-)-epigallocatechin-3-gallate triggered hepatotoxicity in mice: Responses of major antioxidant enzymes and the Nrf2 rescue pathway. Toxicol. Appl. Pharmacol..

[28] Ames M.K., Atkins C.E., Pitt B. (2019). The renin-angiotensin-aldosterone system and its suppression. J. Vet. Intern. Med..

[29] Siragy H.M. (2010). The angiotensin II type 2 receptor and the kidney. J. Renin Angiotensin Aldosterone Syst..

[30] Porrello E.R., Pfleger K.D., Seeber R.M., Qian H., Oro C., Abogadie F., Delbridge L.M., Thomas W.G. (2011). Heteromerization of angiotensin receptors changes trafficking and arrestin recruitment profiles. Cell Signal..

[31] Ryu H.H., Kim H.L., Chung J.H., Lee B.R., Kim T.H., Shin B.C. (2011). Renoprotective effects of green tea extract on renin-angiotensin-aldosterone system in chronic cyclosporine-treated rats. Nephrol. Dial. Transplant..

[32] Siragy H.M., Xue C., Abadir P., Carey R.M. (2005). Angiotensin subtype-2 receptors inhibit renin biosynthesis and angiotensin II formation. Hypertension.

[33] Potthoff S.A., Stamer S., Grave K., Königshausen E., Sivritas S.H., Thieme M., Mori Y., Woznowski M., Rump L.C., Stegbauer J. (2016). Chronic p38 mitogen-activated protein kinase inhibition improves vascular function and remodeling in angiotensin II-dependent hypertension. J. Renin Angiotensin Aldosterone Syst..

